# Patient empowerment in long-term conditions: development and preliminary testing of a new measure

**DOI:** 10.1186/1472-6963-13-263

**Published:** 2013-07-08

**Authors:** Nicola Small, Peter Bower, Carolyn A Chew-Graham, Diane Whalley, Joanne Protheroe

**Affiliations:** 1 Centre for Primary Care, Institute of Population Health, Manchester Academic Health Science Centre, University of Manchester, Oxford Road Williamson Building, Manchester, UK; 2 Research Institute Primary Care and Health Sciences, Keele University, Keele, UK; 3 RTI Health Solutions, The Pavilion, Towers Business Park, Wilmslow Road, Didsbury, Manchester, UK

**Keywords:** Patient empowerment, Long-term conditions, Primary care, Patients’ perspectives, Semi-structured interviews, Measurement, Scale development, Psychometrics, Health outcomes

## Abstract

**Background:**

Patient empowerment is viewed by policy makers and health care practitioners as a mechanism to help patients with long-term conditions better manage their health and achieve better outcomes. However, assessing the role of empowerment is dependent on effective measures of empowerment. Although many measures of empowerment exist, no measure has been developed specifically for patients with long-term conditions in the primary care setting. This study presents preliminary data on the development and validation of such a measure.

**Methods:**

We conducted two empirical studies. Study one was an interview study to understand empowerment from the perspective of patients living with long-term conditions. Qualitative analysis identified dimensions of empowerment, and the qualitative data were used to generate items relating to these dimensions. Study two was a cross-sectional postal study involving patients with different types of long-term conditions recruited from general practices. The survey was conducted to test and validate our new measure of empowerment. Factor analysis and regression were performed to test scale structure, internal consistency and construct validity.

**Results:**

Sixteen predominately elderly patients with different types of long-term conditions described empowerment in terms of 5 dimensions (identity, knowledge and understanding, personal control, personal decision-making, and enabling other patients). One hundred and ninety seven survey responses were received from mainly older white females, with relatively low levels of formal education, with the majority retired from paid work. Almost half of the sample reported cardiovascular, joint or diabetes long-term conditions. Factor analysis identified a three factor solution (positive attitude and sense of control, knowledge and confidence in decision making and enabling others), although the structure lacked clarity. A total empowerment score across all items showed acceptable levels of internal consistency and relationships with other measures were generally supportive of its construct validity.

**Conclusion:**

Initial analyses suggest that the new empowerment measure meets basic psychometric criteria. Reasons concerning the failure to confirm the hypothesized factor structure are discussed alongside further developments of the scale.

## Background

A key feature of current health policy is the focus on long-term conditions (a term used interchangeably with ‘chronic conditions’). These are defined as conditions ‘that cannot be cured but can be managed through medication and/or therapy’ [1]. Policy concerning the management of long-term conditions gives high priority to active *patient participation* in delivery of health care, and to the importance of *self*-*management*[2].

Participation in health care has been defined as ‘an interaction, or series of interactions between a patient and the healthcare system or health care professional in which the patient is active in providing information to aid diagnosis and problem-solving, sharing his/her preferences and priorities for treatment or management, asking questions and/or contributing to the identification of management approaches that best suit his/her needs, preferences or priorities’ [3]. Self-management has been defined as ‘the care taken by individuals towards their own health and well-being: it comprises the actions they take to lead a healthy lifestyle; to meet their social, emotional and psychological needs; to care for their long-term condition; and to prevent further illness or accidents’ [1].

Although there are clearly overlaps between participation and self-management, participation generally refers to patient involvement in decision-making about treatment with their health professional, while self-management is more concerned with health behavior as a result of that decision-making.

### Achieving participation and self-management: the role of empowerment

How are improvements in participation and self-management to be achieved? From a policy perspective, the concept of ‘empowerment’ has been viewed as critical. For example, leading health policy makers in the United Kingdom stated that:

‘*the patient as expert and partner in care is an idea whose time has come*, *and has the potential to create a new generation of patients who are empowered to take action to improve their health in an unprecedented way*’ [4].

The concept of empowerment is used in a wide range of contexts and is generally viewed as a multi-level construct with manifestations at the community, group or individual level [5-7]. At an individual level, empowerment is a process by which individuals experience heightened feelings of control and self-efficacy [8-13]. Conger and Kanungo [8] defined psychological empowerment as a motivational process intertwined with the construct of self-efficacy. Empowerment may result in a re-definition of the roles of and relationships between health care professionals and patients [14-18], and the promotion of greater patient autonomy, with patients making the majority of decisions relating to the care of their conditions [14].

On the basis of this literature and for the purpose of the empirical work, we initially conceptualised empowerment in patients with long-term conditions in primary care as:

*An enabling process or outcome arising from communication with the health care professional and a mutual sharing of resources over information relating to illness*, *which enhances the patient*’*s feelings of control, self-efficacy, coping abilities and ability to achieve change over their condition.*

Therefore, empowerment is a psychological state that occurs as a result of effective communication in health care, and which acts as a determinant of consequent participation and self-management.

Previous studies have shown that patient empowerment in various health settings may be related to: self-reported health [19]; quality of life, social support and self-esteem [20]; education level [21-23]; and current living and work arrangements [20]. There is some empirical evidence to suggest that there is a positive relationship between empowerment and long-term health outcomes [20,24-26].

### The need for a measure of patient empowerment

As empowerment is viewed as a priority by policy makers, patients and professionals, there is consequent interest in improving levels of empowerment [27]. Any systematic attempt to assess empowerment is dependent in part on the effective measurement of the concept.

An unpublished systematic review conducted by the authors found few instruments designed to measure empowerment in patients with long-term conditions and those that exist have been developed for particular long-term conditions, such as diabetes [22], cancer [23] and specific contexts, such as rehabilitation [22] and self-help settings [23]. Instrument development generally involved a mix of literature searches and interviews with patients and professionals [20,22,23,28-32], although few instruments have comprehensive evidence of validity and reliability [23,28-32].

The conceptual models underlying scales and the content of the actual instruments reflect the particular health care context and sample population. For example, the Empowerment Scale was developed for patients with mental health conditions, and the context reflects socio-political concepts around community activism and social action in mental health [20]. In comparison, the Diabetes Empowerment Scale was based on the concept of psychosocial self-efficacy and item content is focused on feelings of confidence and goal setting in self-management [22].

Only one instrument - the Patient Enablement Instrument [33] - was developed to measure empowerment in long-term conditions in primary care, through the associated concept of enablement. However, the measure has some limitations. It was developed to measure empowerment relating to a single consultation only, and was designed to capture the views of patients with a variety of needs (and is not *specific* for patients with long-term conditions). The measure only has 6 items, and although it is highly practical in research and routine settings, the content may not cover the full range of dimensions of empowerment [33].

### Aims of the current study

Primary care is the setting in which a high proportion of patients with long-term conditions are managed [34]. Creating a valid and reliable measure of empowerment for use in this particular setting will assist in exploring the impact of empowerment in primary care and allow the measurement of the effects of interventions which aim to increase empowerment.

The aim of this paper is to report on two empirical studies conducted to understand and measure empowerment in patients with long-term conditions in primary care. Study 1 was a qualitative study which sought to explore the patient and practitioner perspective on empowerment. Thus, we present a summary of that study, with a focus on those patient-related results which directly informed the development of the new measure of empowerment. Study 2 was a quantitative cross-sectional study which provided preliminary testing and validation of the new measure.

## Methods

### Study 1: qualitative study

The purpose of study 1 was to understand empowerment in the management of long-term conditions to assist in developing a conceptual model to inform the measurement of empowerment. A qualitative approach was chosen because little work has been done on understanding the concept of empowerment from the perspective of patients with long-term conditions in primary care.

#### Participants

The study took place within a single Primary Care Trust in the North West of England. Ethical approval was gained from Central Manchester Research Ethics Committee (REC Ref: 08/H1008/159).

The recruitment of patient participants occurred from April to May 2009. Patient participants were sampled from the disease registers of 8 general practices, and sent letters inviting participation. We sampled patients on three registers (diabetes, coronary heart disease (CHD) or asthma) which represent prevalent conditions in primary care, which present common challenges to patients, and include variability in important characteristics such as symptomatology and management. The anticipated sample size was based on previous qualitative research which indicates that category saturation might be achieved within approximately twenty interviews [35].

#### Interviews

Semi-structured, one-to-one interviews were undertaken by the first author between July and October 2009 in patients’ homes. Previous qualitative studies investigating empowerment in patients with specific conditions have favoured using one-to-one interviews over other methods [11,36].

The definition of empowerment described in the introduction was used as a ‘working definition’ to inform the interview topic guide. The definition was broken down into a set of categories representing core concepts, including: communication with health professionals, condition-related information, feelings of control, self-efficacy, coping skills, and ability to achieve change. Questions were specifically formulated to explore each category. For instance, *thinking back, how much did you actually know about this long-term condition before your diagnosis?* was a question used to assess patients’ knowledge of a particular condition before and after diagnosis and to grasp their intentions and health behavior towards managing their condition. The structure of the interview was flexible, allowing for detailed exploration of particular points of interest.

The interviews were audio recorded with consent. Confidentiality was assured at the start of each interview. Demographic characteristics were gathered at the end of the interview.

#### Data analysis

Analysis was guided by a modified grounded theory approach [37]. The justification for using this approach was informed by the qualitative research question, to understand empowerment as experienced from the participants themselves. By following the principles of grounded theory, the researcher could understand participants’ beliefs and attitudes concerning empowerment. Previous instruments have adopted a similar approach as a preliminary step to measuring empowerment [23,28,29].

The initial analysis took an iterative approach [38], with emerging issues from early interviews feeding into future interviews. The data was subjected to a basic thematic analysis and emerging themes coded and categorised accordingly. Deviant cases (that is, those participants who did not follow the emerging analysis) were followed up. Codes were categorized into corresponding families which enabled the formation of sets of concepts amongst the data [38]. All codes were checked by revisiting the transcripts and audio files to assure quality of data. The analysis was carried out with researchers of different professional backgrounds (academic, general practice and psychology) to enhance the reliability of interpretation [39]. The data was coded using the qualitative software package Atlas.ti.

The analysis led to the development of a patient-focused model of empowerment, to assist in the development of a patient instrument.

### Study 2: quantitative study

#### Item generation and pilot testing

Candidate items for each of the 5 dimensions of empowerment that had emerged from study 1 and a literature review (not reported here) were generated by the authors and subjected to an iterative process of development and selection [40]. The wording of items was taken as far as possible from the interview data. Fifty one items were selected from a pool of 60 on the basis of the following criteria: they captured one of the five dimensions of empowerment; reflected a single idea; were unambiguous; and were short in length [41]. A summated self-report Likert scale was selected as an appropriate response format [40].

The measure was subjected to some pilot testing to ensure the candidate items were understandable and acceptable. Two patients with long-term conditions known to the first author were asked to complete and comment on the items. The purpose of these interviews was to simply test the acceptability of the items for patients to complete by post. Members of a local patient and public involvement group (Primary Care Research in Manchester Engagement Resource: PRIMER) were also consulted and gave feedback on the acceptability of the postal questionnaire in terms of wording and formatting. The final list of 51 candidate items for each empowerment outcome is shown in Additional file 1.

#### Validation survey

We conducted a cross-sectional postal survey to provide data for preliminary testing of the reliability and validity of the new measure. Ethical approval for study 2 was gained from Greater Manchester North Ethics Committee (REC Ref: 10/H1011/25).

Following ethical approval, the same practices that participated in study 1 were invited to recruit patients for the survey. Patients with long-term conditions were selected randomly by practice managers from the disease registers for diabetes, asthma, and CHD. General practitioners screened the lists to exclude patients inappropriate for the survey (i.e. those with a recent bereavement or terminal illness). Surveys were mailed out with a reply-paid envelope, with one reminder after two weeks.

A final sample size of 200 was based on current recommendations on case-to-variable ratios in factor analysis and multiple regression [42]. Based on previous surveys in this population [43-45] it was estimated that 33% of patients would respond, therefore 600 respondents were surveyed.

#### Statistical methods

i. ***Scale structure***

As noted earlier, the measure was based on study 1, which suggested that 5 dimensions captured the meaning of empowerment for patients with long-term conditions in primary care. The dimensionality of the new instrument was investigated through exploratory factor analysis, which seeks to reduce a large set of items to a more manageable set of dimensions (or factors).

Following the procedures recommended by Kline [46], we conducted an initial principal components analysis, followed by a scree test to determine the number of factors to rotate. We then conducted a principal axis factor analysis with this number of factors, using an orthogonal rotation to attain a simple factor structure. The factors were interpreted and labeled in relation to the items which loaded on each factor.

ii. ***Reliability***

Internal consistency was assessed by calculating Cronbach’s co-efficient alpha for the dimensions identified by the factor analysis, and for the total scale [47].

iii. ***Construct validity***

Construct validity is defined as the ability of a measure to assess the hypothesized construct (i.e. empowerment). We assessed construct validity through a number of measures where we could hypothesize relationships with overall empowerment (or individual dimensions) based on existing theory or empirical data. Most predictions applied to all dimensions of empowerment, but in some cases predictions were made in relation to specific dimensions. The comparator measures used in predictions are outlined below.

#### Constructed meaning

The Constructed Meaning scale measures the experience of having a long-term condition [48]. The scale has 8 items measured on a 4 point response scale (‘strongly disagree’ to ‘strongly agree’) and has evidence of reliability and validity [48-51].

#### Self efficacy in long-term conditions

The Self Efficacy in Long-Term Conditions scale measures a person’s confidence to perform certain health related activities [52]. The scale has 6 items measured on a 10 point scale (‘not at all confident’ to ‘totally confident’), and has demonstrated reliability and construct validity [53,54]. The scale is frequently used in research in long-term conditions, including our previous research [45,55].

#### Patient assessment of chronic illness care (PACIC)

The PACIC measures the quality of care given to patients in terms of five dimensions of the Chronic Care Model (proactive, planned, patient-centred, problem-solving, and follow-up support [56,57]). The scale has 20 items measured on a 5-point scale (‘almost never’ to ‘almost always’) and has demonstrated evidence of reliability and validity [56].

#### General practice patient survey (GPPS)

The GPPS is a postal survey which assesses patient experience of general practice [44]. To reduce respondent burden, two GPPS questions were used to measure continuity of care and one GPPS question and one GPPS scale were used to measure GP confidence and interpersonal care [44].

The first continuity question assesses GP preference (*is there a particular GP you prefer to see*?) measured by 3 response options (‘there is usually only one doctor in my GP surgery/health centre’, ‘no’ to ‘yes’) and the second continuity question assesses GP continuity (*how often do you see the GP you prefer to see*?) measured by 5 response options (‘not tried at this GP surgery/health centre’, ‘never/almost never, ‘some of the time’, ‘a lot of the time’ to ‘always/almost always’ [44]).

GP confidence was assessed using one question (*When you last saw a GP at your GP surgery about your long*-*term condition*, *did you have confidence and trust in that doctor*?) with four response options (‘don’t know/can’t say’, ‘no, not at all’, ‘yes, to some extent’ to ‘yes, definitely’ [44]). The GP interpersonal care scale has 7 items (*giving you enough time*, *asking about your symptoms*, *listening to you*, *explaining test*, *involving you in decisions*, *treating you with care*, *taking your problems seriously*) measured on a 6 point scale (‘very poor’ to ‘very good’) with a ‘does not apply’ option [44].

The GPPS has been used extensively in postal surveys of general practice populations and has demonstrated evidence of reliability and validity [44,58].

#### Patient enablement instrument (PEI)

The PEI measures a patients’ ability to understand and cope with their condition after seeing their doctor, and the degree to which they feel able to cope with life, keep themselves healthy, feel confident about their health and help themselves [33]. The scale has 6 items measured on a 3 point scale (‘same or less’ to ‘much better’), with a ‘does not apply’ response option. The PEI has demonstrated reliability and construct validity [33,59,60].

#### Patient demographic and clinical characteristics

We also collected patient demographic and clinical characteristics including: number and type of long-term condition(s); length of time since diagnosis; sex; age; ethnicity; education; current work situation; and current living arrangements.

The content of the postal survey can be seen in Additional file 2 and included seven sections (A-G) and 110 questions, including: clinical characteristics (Section A); the new empowerment measure (Section B); 5 comparative measures (Sections C – G), and patient characteristics (Section H).

The associations between empowerment and each comparator measure were evaluated in a series of univariate linear regression models, with empowerment (total scale score) or the individual empowerment dimensions as the dependent variable. Table 1 shows the predicted relationships.

**Table 1:** Hypothesized relationships with empowerment dimensions

**Predictor**	**Predicted relationship with:**		**Supporting empirical and theoretical references**
	***Total score***	***Dimensions***	
Presence of depression as a long-term condition	**-**	***-***	[23,61-63]
Number of long-term conditions	***-***	***-***	[31]
Duration of long-term condition	***+***	***+***	[19,23,24,31,61]
General health	***+***	***+***	[21,23,64]
Identity (Constructed Meaning scale)	***+***	***+ (I)***	[65,66]
Self-efficacy (Self-efficacy in Long-Term Conditions Scale)	***+***	***+***	[22,25,32,67,68]
Patient enablement (PEI)	***+***	***+***	[33,59,69-71]
Quality of chronic care (PACIC)	***+***	***+***	[21,23,63,65,72-75]
Continuity (GPPS)	***+***	***+***	[60,76,77]
Continuity (GPPS preference)	***+***	***+***	[60,78]
Interpersonal care (GPPS)	***+***	***+ (KU)***	[33,75,76,78-80]
Interpersonal care (GPPS confidence)	***+***	***+***	[10,69,76,80-82]
Gender	0	0	[20,21,23,24,32,61,63,65],[74,83,84]
Age	0	0	[21,23,24,61-63,65,85]
Ethnicity	0	0	[20,21,32,65,74,84]
Living arrangements	0	0	[20,21,61]
Education	**+**	**+**	[21-24,31,32,61,83]
Current work	***+***	***+ (KU, EO)***	[20,23,61,74,83]

Note: Identity = Constructed Meaning scale [48]; Self-efficacy = Self-efficacy in Long-Term Conditions scale [52]; PEI Patient Enablement Instrument [33]; PACIC Patient Assessment of Chronic Illness Care [56]; GPPS = General Practice Patient Survey [44]; Continuity (GPPS) and Continuity (GPPS preference) measured using two GPPS questions; Interpersonal care (GPPS) measured using GPPS interpersonal care scale; Interpersonal care (GPPS confidence) measured using a single GPPS question; Predicted relationships: **(+)** Positive relationship; (−**)** Negative relationship; (**0)** No relationship; Tested empirical dimensions: (I) = Identity; (KU) = Knowledge and understanding; (EO) = Enabling others.

## Results

### Study 1: qualitative study

#### Sample characteristics

Three general practices agreed to participate in the study. All three practices were located in relatively deprived areas (with patient list sizes of: 9 250, 8 6143 and 272).

A total of 40 patients were approached and 16 (40%) agreed to participate. Patient interviews lasted between 1 and 3 hours, (shortest 59 minutes, longest 145 minutes). The majority of patients in the sample were older (median 66 years); white (88%); and female (63%). Eighty per cent reported ceasing work due to ill health, or were retired; over three quarters had no educational qualifications; and 50% were home owners. Ten participants had a diagnosis of diabetes; 5 had CHD; and 7 had different respiratory conditions. Altogether, 3 participants had asthma; and 4 participants had either chronic obstructive pulmonary disease (COPD); a lung transplant; emphysema; and silicosis (respiratory conditions listed on the asthma disease register in primary care). Ten participants identified a long-term condition in addition to these diagnoses.

#### Understanding empowerment from the patient perspective

Empowerment in long-term conditions was described by patients as a feeling of control over a long-term condition. The process of empowerment was characterised by an internal process and an external process. The internal process was described by patients as changes in perceptions of the self, following diagnosis. The internal process involved five components: acceptance of the diagnosis; acknowledging the unchangeable; creating a feeling of balance; developing cognitive strategies; and gaining or limiting access to medical information. The external process was a relational process in which support and understanding from friends and practitioners played a central role in empowerment. The external process involved two components: having relational support from significant others and maintaining a relationship with a primary care practitioner.

**Figure 1:**
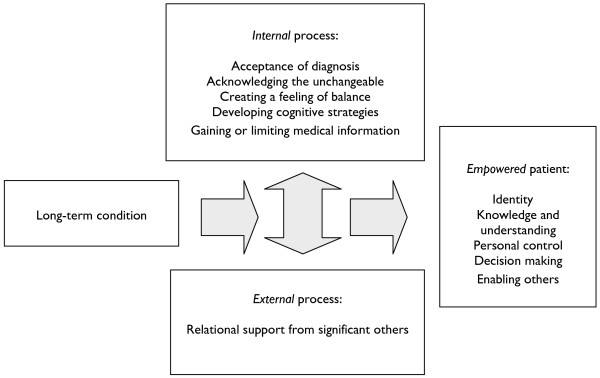
A conceptual model of the process and outcome of empowerment as described by patients’ with long-term conditions in primary care.

Being ‘empowered’ to manage a long-term condition was demonstrated in the data by five measurable outcomes: identity; knowledge and understanding; control; decision making; and enabling other patients with long-term conditions. Figure 1 shows a patient-focused model of empowerment, which assisted the development of a patient instrument.

For the purpose of this paper, we next present data in relation to each of the five dimensions of empowerment, to illustrate the patient-related results which directly informed the development of a new measure of empowerment. Data is presented and identified by participant number and diagnosis of long-term condition.

#### Identity

Empowerment was experienced by patients as changes in perceptions of the self, following diagnosis. For example, participants described how they managed to keep illness as a minor part of life, by minimising the impact on the self and as a result they felt in control.

*I*’*m still working the same. I have the same thoughts and I just carry on. If it hurts stop, have a rest then carry on. But don’t just, I never er just pack up doing a thing. If I can’t do it, I’ll sit down and think of ways around it and explore all avenues. So that’s just the natural thing you know. I don’t know if a lot of people if they got the arterial problem, they’d say ‘oh I won’t do anymore’ well that’s not me, it’s ‘right, I’m getting pain here so let’s see what we do about it, let’s see how much I can do’ work things out you know. But I don’t let things get me down like that. I seem to be good at that. I’m in charge of me self, I control me body and I say you know, you’re going, and we do* (P06, type 2 diabetes/arterial sclerosis).

*I have to accept it is part of my life, it would be foolish to pretend it wasn't, but I try not to let it dominate it, it becomes more a feature of it, like exercise ought to be the part of everybody's life and it is a part of mine. Eating sensibly and healthily ought to be what everybody does and that’s what I am, that's what I try to do you know* (P16, asthma/angina/chronic back pain).

#### Knowledge and understanding

Some participants in the sample purposely limited their knowledge of medical information as a strategy to feel in control, choosing only to have a basic level of knowledge and understanding, and that level was enough for them to manage and feel in control of their conditions.

I: Have you ever looked at any health information online?

R: No.

I: Have you ever thought about it?

*R: No. I think I know enough about me. So that’s the way I work* (P12, type two diabetes/irritable bowel syndrome).

Other participants described feeling in control through a good level of knowledge and understanding of their medical conditions and described having a preference to have information, to feel ‘empowered’ to manage their long-term conditions.

My first port of call probably would be the Internet, because it's the greatest source of knowledge… If I saw something that I would want to look into and check it out I make sure it's valid information… I'd probably go to the internet first and then I would probably go, if I did have a burning issue or anything, then I would email - there is a diabetes nurse - or I'm trying to think who else I know, because I lived in America for a while, and there are Diabetes Educators they call them there (P04, type 1 diabetes).

#### Personal control

Having a perception of personal control in managing a long-term condition outside of the consultation was the third outcome of empowerment. Patients who demonstrated feeling ‘empowered’ described having developed their own personal strategies to stay in control. This was characterised by patients’ engaging in an internal dialogue involving weighing up their thoughts in relation to continuing daily life.

*I don't let it run my life for me. It hasn't really impeded what I do. I don't automatically think ‘I can't do that because I have diabetes’. I am much more likely to think ‘is it possible for me to do that?’ You know, if I fancy doing something, I'll do that. Like as you would do with anything - weigh up the benefits of it against the downside and we all have some sort of, risk management and how we engage with our lives, whether you choose to do something or not* (P14, type 2 diabetes/chronic back pain).

*I take some pills in the morning and some in the afternoon, some after lunch and some in the evening. So I’ve got it spread on different tables. The pills on that table are for the morning, and on another table are for the evening, it’s because I’m on a lot of pills, unfortunately I*’*m on about sixteen a day… when my memory fails me I will have to write it down but so far it’s good just by placing them in different rooms* (P11 type 2 diabetes).

#### Decision making

Being ‘empowered’ was also described by patients as having a feeling to be able to make personal decisions inside the consultation concerning managing their long-term condition and having the choice to participate in the decision making process, including being able to change preferences over time.

I: Have you heard about self-management programmes that you can go to?

R: I've heard of it like, you know, I've got the leaflet from me GP, to go for breathing exercises and all that.

I: Would you ever, is that something that you would ever go to later on, do you think?

*R: Like if I got worse I'd probably go I think because they'd show you how to take inhalers proper, and this and that, and your breathing and all that, well at the moment, I do all that me self* (P02, silicosis/back pain).

*When I go to the lung clinic, if they say something, then I’ll try it and if it doesn’t suit, then I’ll tell them that I can’t do it and that’s the end of it… you can only do so much can’t you* (P07, asthma).

*They [HCPs] packed me off to see a physio and he gave me a series of exercises and I have carried on doing them every single day since, because they help me mobilise and move about. So it is just part of the routine* (P14, type 2 diabetes, chronic/back pain).

#### Enabling others

Some patients also described experiencing sensitivity to others and a desire to motivate or enable others with similar long term conditions to be persistent in coping with their illness.

Furthermore, those patients’ who perceived themselves to be in control of their conditions, felt that they wanted to speak to others with similar conditions, to share their personal experiences of how they managed and by doing so, patients’ described feeling ‘empowered’.

*Because of life’s experience of what I’ve done, people who say ‘I can’t do such a thing’, ‘well have you had a go?’, ‘No’, ‘well how do you know you can’t do it?’, ‘well I don’t think I can do it’, ‘well have you tried?’ ‘well no’, ‘well try it’, ‘well what if I can’t do it?’ ‘Then sit down and think is there a way around it’. You know ‘you keep the brain going’. And ‘what was the problem?’ ‘well it was this…’, ‘right well I have found a way around that, so try such a thing…’* (P06, type 2 diabetes/arterial sclerosis).

Instances of enabling other patients included sharing strategies used to self-manage passing on advice and experience to those coping less well.

*That's me lists of tablets [picks up a ring-binder file containing colour coded charts for each condition] and then that's the daily tablets and that's me stats. I find this works for me, I mean, it shows what I take, so I take that into the Friday club to show um, but I never say, you should do that… but that, that works for me. Some thought it was very good; some thought it looked very difficult* (P09 type 2 diabetes/COPD/recent lung transplant).

### Study 2: quantitative study

#### Sample characteristics

Six hundred surveys were sent out and completed responses were received from 197 people (33%). Characteristics of the survey sample are shown in Table 2.

**Table 2:** Demographic and long-term characteristics of the survey sample

	**Number (%)**
	**Mean (SD)**
	**Range**
*Demographic characteristics:	
Female	102 (52.8)
Age (years)	62.8 (14.3)
	22–88
White	172 (89.7)
No qualifications	71 (37.5)
Retired from paid work	102 (53.1)
Home owner	110 (57.3)
*Long*-*term characteristics*:	
Diabetes	91 (46.2)
Chronic obstructive pulmonary disease	26 (13.2)
Coronary heart disease	33 (16.8)
Irritable bowel syndrome or abdominal (tummy) problems	43 (21.8)
Chronic fatigue syndrome, myalgic encephalomyelitis or fibromyalgia	6 (3.0)
Arthritis or painful joints, back trouble, osteoporosis	103 (52.3)
Heart problems or high blood pressure	104 (52.8)
Anxiety, depression or stress	53 (26.9)
Multiple sclerosis	2 (1.0)
Asthma	31 (15.7)
Other long-term condition (not listed)	49 (24.9)
Number of long-term conditions	2.8 (1.5)
	1–5

Note: *Demographic characteristics that represent our sample from the following categories: Abbreviation; *Female*, Gender; *White*, Ethnic group; *No qualifications*, Educational attainment; *Retired from paid work*, Current work; *Home owner*, Current accommodation.

The majority of patients were older white females, with relatively low levels (38%) of formal education, with the majority (53%) retired from paid work. Almost half of the sample reported cardiovascular and joint conditions and diabetes, and there was evidence of multimorbidity, with over a third of the sample reporting more than one condition.

#### Factor structure

The factor analysis identified 11 components with an eigenvalues greater than 1, but the scree test indicated between 1 and 5 components (n = 197). Principal axis factoring followed by varimax rotation was run using one, three and five factors. The 3 factor solution had the fewest cross-loadings (that is, items that loaded on more than one factor), and explained 45.7% of the total variance (See Table 3). Forty seven of 51 items loaded >0.4. Four items failed to load any of the three factors.

**Table 3:** Factor loadings of the empowerment items (n = 197)

**Empowerment factor label and item (tested empirical dimension)**	**Factor 1**	**Factor 2**	**Factor 3**
*Positive attitude and sense of control*			
I feel useful in my daily life despite my condition (I)	.814		
I feel I have a very good life despite my health problems (I)	.802		
I feel like I am actively involved in life despite my health problems (I)	.790		
I can live a normal life despite my condition (I)	.785		
I am still doing interesting things in my life despite my health problems (I)	.744		
I have plans to do enjoyable things despite my health condition (I)	.739		
*My health problems stop me from enjoying my life (I)	.703		
I have a positive outlook towards my condition (I)	.701		
I find my health problems take over my life (I)	.673		
I feel a sense of control over my condition (PC)	.672		
I have a hopeful outlook towards my condition (I)	.668		
I am capable of handling my condition (PC)	.661		
I can minimise the impact of my symptoms on my life (PC)	.655		
I feel there is purpose and meaning in my life despite my health problems (I)	.623		
*I live my life one day at a time because of my condition (PC)	.581		
I actively manage my condition (PC)	.553		
I am satisfied with my control over the symptoms of my condition (PC)	.553		
Knowing more about my condition helps me to manage it (KU)	.521		
I have the skills that help me feel in control of my condition (PC)	.518		
I try to make the most of my life despite my condition (I)	.493		
Without my health problems I could achieve more (I)	.489		
*Knowledge and confidence in decision making*			
I know enough about my condition (KU)		.747	
I have all the knowledge I need to manage my condition (KU)		.704	
I understand my condition (KU)		.657	
I would feel able to refuse a decision made by my doctor concerning my treatment (DM)		.590	
I know how to handle difficulties related to my condition (PC)		.582	
*I find it difficult to ask my doctor to change my treatment (DM)		.543	
I have information to handle difficulties related to my condition (KU)	.425	.517	
I know how to control my health problems (PC)	.448	.494	
I participate in decisions concerning my health care (DM)		.493	
I am confident choosing among different treatment options related to my condition with my doctor (DM)		.490	.430
I know what my test results mean (KU)		.442	
I know where to go to find something out about my condition (KU)		.426	
I can talk to my doctor if I change my mind concerning my treatment (DM)		.407	
*Enabling others*			
I need to know what is happening to me and why (KU)			.686
I have helped people who have similar conditions find different ways to cope (EO)			.627
I have shared my experience of managing my condition with other people with health problems (EO)			.627
I feel frustrated for other people who are struggling with similar conditions (EO)			.611
I have shared with others how I keep myself well (EO)			.585
I would acquire more health information when needed (KU)			.558
I am aware I can change my mind about a treatment (DM)			.541
I am aware I can choose different treatment options (DM)		.406	.506
*I’m not bothered about understanding health information (KU)			.498
I often request additional health information from my doctor (DM)			487
I would refuse a treatment if I thought it was not the best thing for me (DM)			.458
I have shared my understanding of my condition with people who have similar conditions (EO)			.423
People who are struggling with similar conditions often ask me for advice (EO)			.421

Note: Component extraction method was Principal Axis Factoring, with a cut-point for including factor loadings (>0.4); *Negative worded item; Tested empirical dimensions; (*I*), Identity; (PC), Personal control; (KU), Knowledge and understanding; Four of 51 items failed to load any factor, ‘I am satisfied with the level of health information that I have available to me’ (KU); ‘My own experience has increased my understanding of what it is like for other people to have this condition’ (EO); ‘I accept that I have to live with my condition’ (I); ‘I sometimes take health information to my doctor’ (DM).

The factor analysis did not support the hypothesised five dimensions. The first factor was dominated by items relating to ‘identity’ and ‘personal control’, and was given the preliminary label of ‘positive attitude and sense of control’. The second factor was dominated by items relating to ‘knowledge and understanding’ and ‘decision making’ and was given the preliminary label of ‘knowledge and confidence in decision making’. The third factor was a complex mixture of items relating to ‘enabling others’, ‘knowledge and understanding’ and ‘decision-making’, and there was no obvious label for this factor. We argue that there is a case to restrict factor 3 to those items relating to ‘enabling others’, but accept that the empirical results relating to this factor are less clear, and this initial solution must be considered preliminary.

Given the unclear nature of the three factor solution, we restricted further analyses of reliability and construct validity to the total empowerment scale.

#### Reliability

The alpha of the total empowerment scale was 0.82 and the scale may be considered internally consistent by current conventions e.g. >0.7 [86].

#### Construct validity

The result of the regression models to assess construct validity of the total empowerment scale is shown in Table 4.

**Table 4:** Results of univariate regressions testing predicted and empirical relationships with empowerment total score

**Predictor**	**Predicted relationship with total score**	***β*, 95% CI, P**	**Empirical relationship with total score**
1. Presence of depression as a long-term condition	**-**	−6.98, (−15.92, 1.96), 0.13	0
2. Number of long-term conditions	***-***	−4.96, (−7.95, -1.97)**	***-***
3. Duration of long-term condition(s)	***+***	0.46, (0.07, 0.85)*	***+***
4. General health	***+***	11.65, (7.92, 15.38)**	***+***
5. Identity	***+***	4.64, (3.96, 5.33)**	***+***
6. Self-efficacy	***+***	1.76, (1.44, 2.08)**	***+***
7. PEI	***+***	2.09, (0.85, 3.33)**	***+***
8. PACIC	***+***	0.65, (0.42, 0.87)**	***+***
9. Continuity (GPPS)	***+***	6.49, (−2.87, 15.84), 0.17	0
10. Continuity (GPPS preference)	**+**	22.72, (11.57, 33.86)**	*+*
11. Interpersonal care (GPPS)	***+***	0.91, (0.28, 1.54)**	*+*
12. Interpersonal care (GPPS confidence)	***+***	16.09, (−1.83, 33.99), 0.08	0
13. Gender	0	−0.01, (−0.04, 0.02), 0.49	0
14. Age	0	−0.00, (−0.03, 0.02), 0.51	0
15. Ethnicity	0	15.25, (0.54, 29.97)*	***+***
16. Living arrangements	0	−17.73, (−26.63, -8.83)**	***-***
17. Education	**+**	19.18, (9.83, 28.54)**	**+**
18. Current work	***+***	14.41, (−8.21, 37.03), 0.21	0

Note: Identity, Constructed Meaning scale [48]; Self-efficacy, Self-efficacy in Long-Term Conditions scale [52]; PEI Patient Enablement Instrument [33]; PACIC Patient Assessment of Chronic Illness Care [56]; GPPS General Practice Patient Survey [44]; Continuity (GPPS) and Continuity (GPPS preference) measured using two GPPS questions; Interpersonal care (GPPS) measured using GPPS interpersonal care scale; Interpersonal care (GPPS confidence) measured using a single GPPS question; Summary of results = **(+)** Positive relationship; (−**)** Negative relationship; (**0)** No relationship; *β*, Standardized coefficient of the model; CI, 95% Confidence interval; *P < 0.05; **P < 0.01.

In terms of clinical characteristics, hypothesised relationships were generally supported. A greater number of long-term conditions were associated with lower total empowerment scores. Longer duration of illness and better general health was related to higher total empowerment scores. However, presence of depression as a long-term condition over other long-term conditions was not related to total empowerment scores.

In terms of the psychological factors measured, identity, self-efficacy, and enablement were all related to higher total empowerment scores as predicted.

In terms of process of care, most hypothesised relationships were supported. Perceptions of high quality chronic disease care, seeing a preferred doctor, and perceived quality of interpersonal care were related to higher total empowerment score. Contrary to hypotheses, continuity of care and confidence in the doctor were not related to total empowerment scores.

Finally, in terms of patient demographic characteristics, gender and age were unrelated to total empowerment scores as predicted, except for a significant relationship between ethnicity and lower total empowerment scores. Contrary to hypotheses, current living arrangements were related to lower total empowerment scores. Educational level showed the hypothesised positive relationships with empowerment, but current work was unrelated to total empowerment scores.

## Discussion and conclusions

### Statement of principal findings

The aim of this paper was to report the development and preliminary validation of a new measure of empowerment for patients with long-term conditions in primary care using data from two empirical studies – a qualitative interview study and a cross-sectional quantitative survey.

Being ‘empowered’ to manage a long-term condition was demonstrated in the qualitative data by five dimensions: identity; knowledge and understanding; personal control; decision-making; and enabling other patients with long-term conditions. Questionnaire items were developed in relation to each outcome and tested in a cross-sectional study.

The 5 dimensions identified through conceptual analysis and qualitative work in study 1 were not confirmed empirically in study 2, and a three factor solution was considered to have the simplest structure but this solution was clearly suboptimal and in need of future testing. We applied preliminary labels of ‘positive attitude and sense of control’ and ‘knowledge and confidence in decision making’. The third factor was a complex mixture of items and no clear label was possible.

Given the unclear nature of the three factor solution, we suggest future use of the new measure be restricted to the total empowerment scale, pending future work on the scale structure.

### Strengths and weaknesses of the study

The scale was developed based on a literature review (not reported here) and in-depth qualitative work, thereby enhancing the content validity of the measure. The preliminary pilot testing involved interviews with patients and input from a patient and public involvement group to assess acceptability. However, these sources were known to the author and there may have been a problem of social desirability in their response about item content. We did not use the popular technique of cognitive interviewing to test the items, which would have provided a more rigorous test of comprehension [87].

Test retest reliability of the new scale has yet to be evaluated. The assessment of validity was based on hypotheses regarding associations between the new measure and various comparators. The assessment of validity presented here was based on cross-sectional data and the ability of the scale to predict outcomes longitudinally remains to be demonstrated. A significant number of analyses were conducted, raising issues of multiple hypotheses testing, which might have been avoided through adjustment of the significance level used. However, given the exploratory nature of the work, we wanted to avoid missing any potentially important relationships early into the development process.

All participants were recruited from three general practices and within an inner-city area of North West of England. Sampling was restricted to responders who opted-in to the interview study and on completion of a postal survey. As a result, the respondents in both studies 1 and 2 were relatively homogenous in terms of demographic characteristics (older age, white, retired due to ill health, and located in relatively deprived areas). Ethical requirements meant that respondents had to opt-in to both studies, and such participants may demonstrate certain characteristics, whereas a more diverse sample may have given different results. The potential for bias may be especially high in terms of the limited range of deprivation in the sample, as patients from less deprived backgrounds may have markedly different views.

The response rate was relatively low in study 2, although largely in line with current surveys in this population locally [43] and nationally [58]. However, it should be noted that the study was not designed to assess prevalence, and that response bias will likely have more limited effects on assessment of relationships between variables (although it may lead to restriction in range if certain types of patients do not respond). It was not possible to explore characteristics of non-respondents as current ethical guidelines do not allow data recording on patients who do not consent to participate.

### Interpretation of the results

As outlined previously, the existence of five distinct dimensions of empowerment suggested by the qualitative work were not supported by the quantitative findings in study 2. The factor analyses suggested that responses to items concerning ‘identity’ and ‘control’ were related, as were issues of ‘knowledge and understanding’ and ‘decision making’, and these relationships make conceptual sense.

The meaning of the third factor was very unclear, but the fact that items related to ‘enabling others’ only loaded on this factor might suggest that further tests using only these items would be useful, to see if the validity of this factor was supported. The factor was derived from the patient interviews, and is interesting as it relates to current self-management initiatives in the NHS, such as the Expert Patients Programme [88], and the Health Trainers initiative [89], which both rely on non-professionals to teach and empower patients who may be coping less well with their long-term conditions [46]. We would suggest that further psychometric work on this scale is indicated as it has potential wider utility.

Given the ambiguities over the factor solution, we restricted our initial analyses of construct validity to the total empowerment score. In terms of construct validity, the majority of hypotheses were supported from the regression results. Seeing a preferred GP, being educationally qualified and general health were strong predictors of increases in total empowerment. Hypotheses that were not confirmed included the importance of continuity of care and GP confidence and total empowerment.

It is noteworthy that seeing a preferred GP emerged as a key predictor in the analysis, but continuity with the doctor and GP confidence were found to be weak predictors of empowerment. It should be noted that nearly half (49.8%) of responders had not seen their GP/practice nurse for at least 7 to 9 months, which may have introduced a bias in responses on empowerment items that related to GP variables. Only two aspects of continuity were included in this study: seeing the same GP and seeing a preferred GP. It is possible that other measures of continuity may have given different results. For instance, twelve continuity measures have been developed to measure various types of continuity [90], each emphasizing different elements of the patient-practitioner relationship, such as density of visits and subjective perception of visit [91]. It may also reflect the fact that nurses provide the bulk of long-term condition care in the United Kingdom.

### Future research

Questionnaire development and validation is an ongoing process, and there are a number of potential developments of the proposed new empowerment measure.

Future testing of the measure may also benefit from confirmatory factor analysis to more rigorously test hypotheses about scale structure, given that ambiguities remain concerning the validity of the three factor solution and the concern over scale length.

We are aware routine use of the new measure is likely to be enhanced by reducing the number of items. Following future large-scale validation of the measure, a short-form version should be developed and tested to lower response burden and increase the possibility of routine use of the measure. The development and testing of the short-version should follow state of the art methodology for shortening composite measurement scales [92].

If additional supportive evidence is generated, the scale might usefully be used in longitudinal studies or randomised trials of self-care interventions, to see whether it is sensitive to the effects of interventions designed to improve empowerment, and whether such changes are subsequently associated with changes in self-care behaviours, health outcomes and quality of life. Interventions that might be expected to lead to increased empowerment might include: GP communication skills training [93]; patient decision aid interventions [94]; and self-management support, such as the chronic disease self-management programme [45].

Future research might also explore the relationship between measures of illness perceptions, such as the Illness Perception Questionnaire [95], which is designed to capture representations of specific illnesses, rather than generic feelings of empowerment. Exploring relationships between the measures may be fruitful, as it is possible that certain types of illness representations (such as those around controllability) may be predictive of levels of empowerment.

The new measure has similarities with the Patient Activation Measure [96] and a formal comparison might highlight advantages and disadvantages of each. Both measures have different psychometric properties and underlying scale structure. The Patient Activation Measure [96] has stronger psychometric properties than the current measure and was developed from the Rasch Rating Scale Model [97], an alternative statistical method to factor analysis, used to test scale structure. As a result, the elements of knowledge, belief, and skill that constitute activation have a hierarchical order; thus what is needed to increase activation depends on where the person is on the activation continuum.

There are many other factors not measured in the current study that could be used to assess construct validity. For instance, a quality of life measure or other measures of psychological functioning may have been useful. It was also evident from the qualitative data (not presented here), that health literacy was a key issue in this group of patients, and this could have been examined by administering a health literacy measure [98]. However, such scales can be difficult to use in the context of a postal survey.

## Conclusion

Our preliminary validation study suggests that the proposed empowerment measure meets basic psychometric criteria in terms of reliability and validity, although ambiguities remain about the structure of the scale. A more comprehensive assessment of the psychometric quality of the scale is required, to assess its utility as an outcome measure and as a possible research tool to assess the complex relationships between patient characteristics, the delivery of health care, empowerment, and important outcomes such as self-care, quality of life and health care costs.

Should further work confirm the validity of the empowerment measure, it may have utility in capturing aspects of health from the patient perspective to assess the quality of NHS services. Our measure may be useful to ensure that empowerment as a measurable outcome, receives attention alongside other more clinically focused outcome measures.

## Competing interests

The authors declare that they have no competing interests.

## Authors’ contributions

This doctoral study was conducted by NS. All authors were involved in the development of the measure. NS managed the study and conducted the factor analysis and regressions. NS, PB, JP, CCG and DW wrote the paper. All authors read and approved the final manuscript.

## Pre-publication history

The pre-publication history for this paper can be accessed here:

http://www.biomedcentral.com/1472-6963/13/263/prepub

## Supplementary Material

Additional file 1 Fifty one candidate items with corresponding empowerment dimension. Click here for file

Additional file 2 The postal survey completed by patients in this study. Click here for file

## References

[B1] Department of Health Self care: a national view in 2007 compared to 2004–2005 2007 London: Stationery Office

[B2] Hibbard J Collins P Mahoney E Baker L The development and testing of a measure assessing clinician beliefs about patient self-management Health Expect 2009 13 65 72 19906211 10.1111/j.1369-7625.2009.00571.x PMC5060511

[B3] Haywood K Marshall S Fitzpatrick R Patient participation in the consultation process: a structured review of intervention strategies Pat Educ Couns 2006 63 12 23 10.1016/j.pec.2005.10.005 16406464

[B4] Donaldson L Expert patients usher in a new era of opportunity for the NHS BMJ 2003 326 1279 1280 10.1136/bmj.326.7402.1279 12805129 PMC1126164

[B5] Zimmerman MA Rappaport J Citizen participation, perceived control and psychological empowerment American J Comm Psychol 1988 16 725 750 10.1007/BF00930023 3218639

[B6] Zimmerman MA Psychological empowerment: issues and illustrations J Comm Psychol 1995 23 581 599 10.1007/BF02506983 8851341

[B7] Roberts KJ Patient empowerment in the United States: a critical commentary Health Expect 1999 2 82 92 10.1046/j.1369-6513.1999.00048.x 11281882 PMC5061436

[B8] Conger J Kanungo R The empowerment process: integrating theory and practice Acad Man Rev 1988 13 471 482

[B9] Zimmerman MA Toward a theory of learned hopelessness: a structural model of analysis of participation and empowerment J Res Pers 1990 24 71 86 10.1016/0092-6566(90)90007-S

[B10] Gibson CH A concept analysis of empowerment J Adv Nurs 1991 16 354 361 10.1111/j.1365-2648.1991.tb01660.x 2037742

[B11] Gibson CH The process of empowerment in mothers of chronically ill children J Adv Nurs 1995 21 1201 1210 10.1046/j.1365-2648.1995.21061201.x 7665789

[B12] Segal SP Silverman C Temkin T Measuring empowerment in client-run self-help agencies Comm Mental Health J 1995 31 215 227 10.1007/BF02188748 7621659

[B13] Aujoulat I Luminet O Deccache A The perspective of patients on their experience of powerlessness Qual Health Res 2007 17 772 785 10.1177/1049732307302665 17582020

[B14] Feste CC A practical look at patient empowerment Diab Care 1992 15 922 925 1516517

[B15] Samuel-Hodge C DeVellis R Ammerman A Keyserling T Reliability and validity of a measure of perceived diabetes and dietary competence in African American women with type 2 diabetes Diab Educat 2002 26 979 988 10.1177/014572170202800612 12526638

[B16] Funnell MM Anderson RM Patient empowerment: a look back, a look ahead Diab Educat 2003 29 454 464 10.1177/014572170302900310 12854337

[B17] Funnell MM Anderson RM Empowerment and self-management of diabetes Clinic Diab 2004 22 123 127 10.2337/diaclin.22.3.123

[B18] Anderson RM Funnell MM Barr PA Dedrick RF Davis WK Learning to empower patients: results of professional education program for diabetes educators Diab Care 1991 14 584 590 10.2337/diacare.14.7.584 1914799

[B19] Leksell J Funnell M Sandberg G Smide B Wiklund G Wikblad K Psychometric properties of the Swedish Diabetes Empowerment Scale Scand J Caring Sci 2007 21 247 252 10.1111/j.1471-6712.2007.00463.x 17559444

[B20] Rogers ES Chamberlin J Ellison ML Crean T A consumer-constructed scale to measure empowerment among users of mental health services Psychiat Serv 1997 48 1042 1047 10.1176/ps.48.8.1042 9255837

[B21] Sigurdardottir A Jonsdottir H Empowerment in diabetes care: towards measuring empowerment Scand J Caring Sci 2008 22 284 291 10.1111/j.1471-6712.2007.00506.x 18298619

[B22] Anderson RM Funnell MM Fitzgerald JT Marrero DG The Diabetes Empowerment Scale: a measure of psychosocial self-efficacy Diab Care 2000 23 739 743 10.2337/diacare.23.6.739 10840988

[B23] Suen SM A model of empowerment for Hong Kong Chinese cancer patients and the role of self-help groups in the empowering process (Hong Kong Polytechnic (People’s Republic of China)) PhD 1998 33123861094

[B24] Atak N Kose K Gurkan T The impact of patient education on Diabetes Empowerment Scale (DES) and Diabetes Attitude Scale (DAS-3) in patients with type 2 diabetes Turk J Med Sci 2008 38 49 57

[B25] Shiu ATY Li S Thompson DR The concurrent validity of the Chinese version of the Diabetes Empowerment Scale Diab Care 2005 28 498 499 10.2337/diacare.28.2.498-a 15677827

[B26] Anderson RM Funnell MM Nwankwo R Gillard ML Oh M Fitzgerald JT Evaluating a problem-based empowerment program for African Americans with diabetes: results of a randomized controlled trial Ethn Dis 2005 15 671 678 16259492

[B27] Department of Health Equity and excellence: liberating the NHS 2010 London: Stationery Office

[B28] Bulsara C Styles I Ward AM Bulsara M The psychometrics of developing the Patient Empowerment Scale J Psychosoc Oncol 2006 24 1 16 17046803 10.1300/J077v24n02_01

[B29] Klima C Vonderheid S Norr K Measuring empowerment in pregnancy: the Pregnancy-Related Empowerment Scale… Conference proceedings: abstracts from Research Forums presented at the ACNM 52nd Annual Meeting 2007 J Mid Women’s Health 2007 34 53123871886

[B30] Faulkner M A measure of patient empowerment in hospital environments catering for older people J Adv Nurs 2001 34 676 86 10.1046/j.1365-2648.2001.01797.x 11380736

[B31] Mikky IF Development of the Client Empowerment Scale (CES) (University of Connecticut) PhD 2006 165

[B32] Vigil BA Development and validation of a measure of empowerment for individuals suffering from eating problems Diss Abstracts Internat 2006 66 4531

[B33] Howie J Heaney D Maxwell M Measuring quality in general practice Occasional Paper Series 1997 75 PMC2560570 9141884

[B34] Beaglehole R Epping-Jordan J Patel V Chopra M Ebrahim S Kidd M Improving the prevention and management of chronic disease in low-income and middle-income countries: a priority for primary health care Lancet 2008 372 940 949 10.1016/S0140-6736(08)61404-X 18790317

[B35] Blakeman T Chew-Graham C Reeves D Rogers A Bower P The Quality and Outcomes Framework and self-management dialogue in primary care consultations: a qualitative study BJGP 2011 61 666 673 10.3399/bjgp11X601389 PMC3177136 22152849

[B36] Wahlin I Ek AC Idvall E Patient empowerment in intensive care: an interview study Intens Crit Care Nurs 2006 22 370 377 10.1016/j.iccn.2006.05.003 16890438

[B37] Glaser B Theoretical Sensitivity 1978 Mill Valley: Sociology Press

[B38] Miles M Huberman A Qualitative data analysis 1994 2 London: Sage

[B39] Henwood K Pidgeon N Qualitative research and psychological theorizing British J Psychol 1992 83 97 111 10.1111/j.2044-8295.1992.tb02426.x 1559146

[B40] DeVellis R Scale development: theory and applications 2003 2 London: Sage

[B41] Costello A Osborne J Best practices in exploratory factor analysis: four recommendations for getting the most from your analysis Prac Assess Res Eval 2005 10 1 9

[B42] Tabachnick B Fidell L Multiple regression Using multivariate statistics 1996 New York: HarperCollins College Publishers 127 193

[B43] Bower P Kennedy A Reeves D Rogers A Blakeman T Chew-Graham C A cluster randomised controlled trial of the clinical and cost-effectiveness of a ‘whole systems’ model of self-management support for the management of long-term conditions in primary care: trial protocol Implement Science 2012 7 1 13 10.1186/1748-5908-7-1 PMC3274470 22280501

[B44] Campbell J Smith P Nissen S Bower P Elliott M Roland M The GP Patient Survey for use in primary care in the National Health Service in the UK - development and psychometric characteristics BMC Fam Pract 2009 10 57 10.1186/1471-2296-10-57 19698140 PMC2736918

[B45] Kennedy A Reeves D Bower P Lee V Middleton E Richardson G The effectiveness and cost effectiveness of a national lay led self care support programme for patients with long-term conditions: a pragmatic randomised controlled trial J Epidemiol Comm Health 2007 61 254 261 10.1136/jech.2006.053538 PMC2652924 17325405

[B46] Kline P Principal components analysis An Easy Guide to Factor Analysis 1994 London and New York: Routledge 28 41

[B47] Cronbach L Cronbach alpha and the internal structure of tests Psychometrika 1951 16 297 334 10.1007/BF02310555

[B48] Fife BL The measurement of meaning in illness Soc Sci Med 1995 40 1021 1028 10.1016/0277-9536(94)00174-R 7597456

[B49] Fife B Scott L Fineberg N Zwickl B Promoting adaptive coping by persons with HIV disease: evaluation of a patient/partner intervention model J Assoc Nurs AIDS Care 2008 19 75 84 10.1016/j.jana.2007.11.002 PMC2743914 18191771

[B50] Fife B The role of constructed meaning in adaptation to the onset of life-threatening illness Soc Sci Med 2005 61 2132 2143 10.1016/j.socscimed.2005.04.026 16026913

[B51] Bevvino D Sharkin B Divorce adjustment as a function of finding meaning and gender differences J Divorce Remarriage 2003 39 81 97 10.1300/J087v39n03_04

[B52] Lorig K Stewart A Ritter P Gonzalez V Laurent D Lynch J Outcome measures for health education and other healthcare interventions 1996 London: SAGE Publications

[B53] Lorig K Sobel D Ritter P Laurent D Hobbs M Effect of a self-management program for patients with chronic disease Effect Clin Pract 2001 4 256 262 11769298

[B54] Marks R Allegrante JP Lorig K A review and synthesis of research evidence for self-efficacy-enhancing interventions for reducing chronic disability: implications for health education practice (Part II) Health Promot Pract 2005 6 148 156 10.1177/1524839904266792 15855284

[B55] Kennedy A Rogers A Bower P Support for self care for patients with chronic disease BMJ 2007 335 968 970 10.1136/bmj.39372.540903.94 17991978 PMC2071971

[B56] Glasgow R Wagner E Schaefer J Mahoney L Reid R Greene S Development and validation of the Patient Assessment of Chronic Illness Care (PACIC) Med Care 2005 43 436 444 10.1097/01.mlr.0000160375.47920.8c 15838407

[B57] Wagner E Austin B Von Korff M Organizing care for patients with chronic illness Milbank Q 1996 74 511 544 10.2307/3350391 8941260

[B58] Roland M Elliott M Lyratzopoulos G Barbiere J Parker R Smith P Reliability of patient responses in pay for performance schemes: analysis of National General Practitioner Patient Survey data in England BMJ 2009 339 1 6 10.1136/bmj.b3851 PMC2754504 19808811

[B59] Howie J Heaney D Maxwell M Walker J A comparison of Patient Enablement Instrument (PEI) against two established satisfaction scales as an outcome measure of primary care consultations Fam Pract 1998 15 165 171 10.1093/fampra/15.2.165 9613486

[B60] Howie JGR Heaney DJ Maxwell M Walker JJ Freeman GK Rai H Quality at general practice consultations: cross sectional survey BMJ 1999 319 738 743 10.1136/bmj.319.7212.738 10487999 PMC28226

[B61] Hansson L Bjorkman T Empowerment in people with a mental illness: reliability and validity of the Swedish version of an empowerment scale Scand J Caring Sci 2005 19 32 38 10.1111/j.1471-6712.2004.00310.x 15737163

[B62] Shiu A Martin C Thompson D Wong R Psychometric properties of the Chinese version of the Diabetes Empowerment Scale Psychol Health Med 2006 11 198 208 10.1080/13548500500286845 17129908

[B63] Lloyd C King R Moore L Subjective and objective indicators of recovery in severe mental illness: a cross-sectional study Int J Soc Psychiat 2009 24 425 434 10.1177/0020764009105703 19592440

[B64] Leskell J Funnell M Sandberg G Smide B Wiklund G Wikblad K Psychometric properties of the Swedish Empowerment Scale Scand J Caring Sci 2007 21 247 252 10.1111/j.1471-6712.2007.00463.x 17559444

[B65] Strack KM Understanding empowerment, meaning, and perceived coercion in individuals with serious mental illness J Clin Psychol 2009 65 1137 1148 10.1002/jclp.20607 19670431

[B66] Peterson R Grippo K Tantleff-Dunn S Empowerment and powerlessness: a closer look at the relationship between feminism, body image and eating disturbance Sex Roles 2008 58 639 648 10.1007/s11199-007-9377-z

[B67] Vauth R Kleim B Wirtz M Corrigan P Self-efficacy and empowerment as outcomes of self-stigmatizing and coping in schizophrenia Psychiat Res 2007 150 71 80 10.1016/j.psychres.2006.07.005 17270279

[B68] Tsay S Hung L Empowerment of patients with end-stage renal disease- a randomized controlled trial Int J Nurs Stud 2004 41 59 65 10.1016/S0020-7489(03)00095-6 14670395

[B69] Rodwell C An analysis of the concept of empowerment J Adv Nurs 1996 23 305 313 10.1111/j.1365-2648.1996.tb02672.x 8708244

[B70] Hokanson Hawks J Empowerment in nursing education: concept analysis and application to philosophy, learning and instruction J Adv Nurs 1992 17 609 618 10.1111/j.1365-2648.1992.tb02840.x 1602078

[B71] Ellis-Stoll C Popkess-Vawter S A concept analysis on the process of empowerment Adv Nurs Sci 1998 21 62 68 10.1097/00012272-199812000-00007 9845487

[B72] Jormfeldt H Arvidsson B Svensson B Hansson L Construct validity of a health questionnaire intended to measure the subjective experience of health among patients in mental health services J Psychiat Mental Health Nurs 2008 15 238 245 10.1111/j.1365-2850.2007.01219.x 18307653

[B73] Chiba R Miyamoto Y Kawakami N Reliability and validity of the Japanese version of the Recovery Scale (RAS) for people with chronic mental illness: scale development Int J Nurs Stud 2010 47 314 322 10.1016/j.ijnurstu.2009.07.006 19679304

[B74] Corrigan PW Faber D Rashid F Leary M The construct validity of empowerment among consumers of mental health services Schizo Res 1999 38 77 84 10.1016/S0920-9964(98)00180-7 10427613

[B75] Svedberg P Svensson B Arvidsson B Hansson L The construct validity of a self-report questionnaire focusing on health promotion interventions in mental health services J Psychiat Mental Health Nurs 2007 14 566 572 10.1111/j.1365-2850.2007.01129.x 17718729

[B76] Abdoli S Ashktorab T Ahmadi F Parvizi S Dunning T The empowerment process in people with diabetes: an Iranian perspective Int Nurs Rev 2008 55 447 453 10.1111/j.1466-7657.2008.00664.x 19146557

[B77] Freeman G Walker J Heaney D Howie J Personal continuity and the quality of GP consultations Euro J Gen Pract 2002 8 90 4 10.3109/13814780209160846

[B78] Little P Everitt H Williamson I Warner G Moore M Gould C Observational study of effect of patient centredness and positive approach on outcomes of general practice consultations BMJ 2001 323 908 911 10.1136/bmj.323.7318.908 11668137 PMC58543

[B79] Swarbrick M Schmidt L Pratt C Consumer-operated self-help centers: environment, empowerment, and satisfaction J Psychosoc Nurs Mental Health Serv 2009 47 40 47 10.3928/02793695-20090527-03 19678478

[B80] Holmstrom I Roing M The relation between patient-centeredness and patient empowerment: a discussion on concepts Pat Educ Couns 2010 79 167 172 10.1016/j.pec.2009.08.008 19748203

[B81] Nyatanga L Dann L Empowerment in nursing: the role of philosophical and psychological factors Nurs Philos 2002 3 234 239 10.1046/j.1466-769X.2002.00107.x

[B82] Aujoulat I d'Hoore W Deccache A Patient empowerment in theory and practice: polysemy or cacophony? Pat Educ Couns 2007 66 13 20 10.1016/j.pec.2006.09.008 17084059

[B83] Wowra S McCarter R Validation of the Empowerment Scale with an outpatient mental health population Psyciat Serv 1999 50 959 961 10.1176/ps.50.7.959 10402621

[B84] Corrigan PW Impact of consumer-operated services on empowerment and recovery of people with psychiatric disabilities Psychiat Serv 2006 57 1493 1496 10.1176/appi.ps.57.10.1493 17035571

[B85] Viklund G Ortqvist E Wikblad K Assessment of an empowerment education programme: a randomized study in teenagers with diabetes Diab Med 2007 24 550 556 10.1111/j.1464-5491.2007.02114.x 17367306

[B86] Nunnally J Psychometric theory 1978 2 New York: McGraw-Hill

[B87] Beatty PC Willis GB Research synthesis: the practice of cognitive interviewing Pub Opinion Quart 2007 71 287 311 10.1093/poq/nfm006

[B88] Department of Health Supporting people with long-term conditions: an NHS and social care model to support local innovation and integration 2005 London: Stationery Office

[B89] Department of Health Choosing health: making healthy choices easier 2004 London: Stationery Office

[B90] Adler R Vasiliadis A Bickell N The relationship between continuity and patient satisfaction: a systematic review Fam Pract 2010 27 171 178 10.1093/fampra/cmp099 20053674

[B91] Jee S Cabana M Indices for continuity of care: a systematic review of the literature Med Care Res Rev 2006 63 158 188 10.1177/1077558705285294 16595410

[B92] Goetz C Coste J Lemetayer F Rat A Montel S Recchia S Item reduction based on rigorous methodological guidelines is necessary to maintain validity when shortening composite measurement scales J Clin Epidemiol 2013 66 710 718 10.1016/j.jclinepi.2012.12.015 23566375

[B93] Bonvicini KA Impact of communication training on physician expression of empathy in patient encounters Pat Edu Couns 2009 75 3 10 10.1016/j.pec.2008.09.007 19081704

[B94] Protheroe J Bower P Chew-Graham C The use of mixed methodology in evaluating complex interventions: identifying patient factors that moderate the effects of a decision aid Fam Pract 2007 24 594 600 10.1093/fampra/cmm066 18039724

[B95] Weinman J Petrie K Moss-Morris R Horne R The Illness Perception Questionnaire: a new method for assessing illness perceptions Psychol Health 1996 11 431 445 10.1080/08870449608400270

[B96] Hibbard J Stockard J Mahoney E Development of the Patient Activation Measure (PAM): conceptualizing and measuring activation in patients and consumers HSR 2004 39 1005 1026 15230939 10.1111/j.1475-6773.2004.00269.x PMC1361049

[B97] Rasch G Probabalistic models for some intelligence and attainment tests 1980 Chicago: University of Chicago Press

[B98] Jordan JE Buchbinder R Osborne RH Conceptualising health literacy from the patient perspective Pat Edu Couns 2010 79 36 42 10.1016/j.pec.2009.10.001 19896320

