# Supplementary material for: Patient empowerment in long-term conditions: development and preliminary testing of a new measure
Source: BMC Health Serv Res. 2013 Jul 8;13:263. doi: 10.1186/1472-6963-13-263 (PMC3725177; doi:10.1186/1472-6963-13-263)
Supplement: Additional file 1 — Fifty one candidate items with corresponding empowerment dimension. [file 1472-6963-13-263-S1.docx]

**Additional File 1** Fifty one candidate empowerment items with corresponding empowerment dimension.

| **Item content** | **Dimension** |
| --- | --- |
| 1. *I have a positive outlook towards my condition* 2. *I feel useful in my daily life despite my condition* 3. *I accept that I have to live with my condition* 4. ***My health problems stop me from enjoying life*** 5. *I feel I have a very good life despite my health problems* 6. *I am still doing interesting things in my life despite my health problems* 7. *I try to make the most of my life despite my condition* 8. *I have plans to do enjoyable things despite my health problems* 9. *I feel like I am actively involved in life despite my health problems* 10. *I feel there is a purpose and meaning in my life despite my health problems* 11. *I have a hopeful outlook towards my condition* 12. *I can live a normal life despite my condition* 13. *I find my health problems take over my life* 14. ***Without my health problems I could achieve more*** | I |
| 1. *Knowing more about my illness helps me to manage it* 2. *I know enough about my condition* 3. *I understand my condition* 4. *I have all the knowledge I need to manage my condition* 5. *I have information to handle difficulties related to my condition* 6. *I know what my test results mean* 7. *I often request additional health information from my doctor* 8. ***I’m not bothered about understanding health information*** 9. *I know where to go to find something out about my condition* 10. *I am satisfied with the level of health care information that I have available to me* 11. *I would acquire more health information when needed* 12. *I need to know why what is happening to me* | KU |

Note: Items in bold are negatively worded items; Dimensions: (I) = Identity; (KU) = Knowledge and understanding.

**Additional File 1** Fifty one candidate empowerment items with corresponding empowerment dimension.

| **Item content** | **Dimension** |
| --- | --- |
| 1. *I am capable of handling my condition* 2. *I feel a sense of control over my condition* 3. *I know how to handle difficulties related to my condition* 4. *I can minimise the impact of my symptoms on my life* 5. *I have the skills that help me to feel in control of my condition* 6. *I know how to control my health problems* 7. *I actively manage my condition* 8. *I am satisfied with my control over the symptoms of my condition* 9. ***I live my life one day at a time because of my condition*** | PC |
| 1. *I participate in decisions concerning my health care* 2. *I am aware I can choose different treatment options* 3. *I would refuse a treatment if I thought it was not the best thing for me* 4. *I sometimes take health information that I have found to my doctor* 5. *I am aware I can change my mind about a treatment* 6. *I can talk to my doctor if I change my mind concerning my treatment* 7. ***I find it difficult to ask my doctor to change my treatment*** 8. *I am confident choosing among different options related to my condition with my doctor* 9. *I would feel able to refuse a decision made by my doctor concerning my treatment* | DM |
| 1. *I have helped people who have similar conditions find different ways to cope* 2. *I have shared with others how I keep myself well* 3. *I have shared my experience of managing my condition with other people with health problems* 4. *People who are struggling with similar health conditions ask me for advice* 5. *My own experience has increased my understanding of what it is like for other people to have this condition* 6. *I feel frustrated with other people who are coping less well with similar conditions* 7. *I have shared my understanding with people who are coping less well with similar conditions* | EO |

Note: Items in **bold** are negatively worded items; Dimensions: (PC) = Personal control; (DM) = Decision making; (EO) = Enabling others.
