# Supplementary material for: Patient empowerment in long-term conditions: development and preliminary testing of a new measure
Source: BMC Health Serv Res. 2013 Jul 8;13:263. doi: 10.1186/1472-6963-13-263 (PMC3725177; doi:10.1186/1472-6963-13-263)
Supplement: Additional file 2 — The postal survey completed by patients in this study. [file 1472-6963-13-263-S2.pdf]

Additional file 2 The postal survey completed by the patients in this study.

National Primary Care Research & Development Centre

MANCHESTER 1824 The University of Manchester

Living with Long-term Conditions

Dear Sir or Madam,

The National Primary Care Research and Development Centre at the University of Manchester is funding a study looking at the experience of patients living with long- term conditions. Long- term conditions include a range of problems like diabetes, arthritis and heart disease.

Our aim is to develop a questionnaire that captures how you manage your condition, and how you understand your health and wellbeing. **Your GP fully supports this study.**

We would be grateful if you would complete this survey and return it in the prepaid envelope provided (no stamp is required). Some of the questions may seem quite similar. This is because we are trying to find the best way of asking certain questions and we would like your help in this process.

Please answer ALL the questions. **Your answers are very important to us.** There are no right or wrong answers, so please just read each question carefully and be as honest as you can.

All the data is completely anonymous and your name and address will not be used.

If you have any problems completing the form, or you would like further information about this study, please contact me on 0161 275 0741 and I will be happy to phone you back.

Many thanks for your time and help,

Researcher
National Primary Care Research and Development Centre
5th Floor, Williamson Building
University of Manchester, Oxford Road
Manchester, M13 9PL
Tel: 0161 275 0741
E-mail: Nicola.small@manchester.ac.uk

A. Your long-term health condition(s)...

1. What long term health condition(s) do you have?

(Please tick **all** the appropriate boxes which include any condition you have)

1. Diabetes	☐	1
2. Chronic obstructive pulmonary disease (COPD)	☐	2
3. Coronary heart disease (CHD)	☐	3
4. Irritable bowel syndrome (IBS) or abdominal (tummy) problems	☐	4
5. Chronic fatigue syndrome, myalgic encephalomyelitis (ME) or fibromyalgia	☐	5
6. Arthritis or painful joints, back trouble, osteoporosis	☐	6
7. Heart problems or high blood pressure	☐	7
8. Anxiety, depression or stress	☐	8
9. Multiple sclerosis	☐	9
10. Other conditions (Please give details)	☐	10

....

....

Please answer the following questions in terms of your long term condition(s)

B. About your beliefs, attitudes and feelings ...

Please read each statement carefully and then indicate the response that best describes the extent to which you agree or disagree in relation to your long-term condition(s)

	Strongly Disagree	Disagree	Neither	Agree	Strongly Agree
1. I often request additional health information from my doctor	☐1	☐2	☐3	☐4	☐5
2. I am aware I can change my mind about a treatment	☐1	☐2	☐3	☐4	☐5
3. I actively manage my condition	☐1	☐2	☐3	☐4	☐5
4. I am still doing interesting things in my life despite my health problems	☐1	☐2	☐3	☐4	☐5
5. I feel frustrated for other people who are struggling with similar conditions	☐1	☐2	☐3	☐4	☐5
6. I am capable of handling my condition	☐1	☐2	☐3	☐4	☐5
7. I am aware I can choose different treatment options	☐1	☐2	☐3	☐4	☐5
8. I am satisfied with the level of health care information that I have available to me	☐1	☐2	☐3	☐4	☐5
9. I'm not bothered about understanding health information	☐1	☐2	☐3	☐4	☐5
10. I am satisfied with my control over the symptoms of my condition	☐1	☐2	☐3	☐4	☐5
11. I have a hopeful outlook towards my condition	☐1	☐2	☐3	☐4	☐5

	Strongly Disagree	Disagree	Neither	Agree	Strongly Agree
12. I have helped people who have similar conditions find different ways to cope	☐1	☐2	☐3	☐4	☐5
13. I would acquire more health information when needed	☐1	☐2	☐3	☐4	☐5
14. My heath problems stop me from enjoying life	☐1	☐2	☐3	☐4	☐5
15. I can minimise the impact of my symptoms on my life	☐1	☐2	☐3	☐4	☐5
16. I find it difficult to ask my doctor to change my treatment	☐1	☐2	☐3	☐4	☐5
17. I have shared my experience of managing my conditions with other people with health problems	☐1	☐2	☐3	☐4	☐5
18. I know where to go to find something out about my condition	☐1	☐2	☐3	☐4	☐5
19. I have plans to do enjoyable things despite my health condition	☐1	☐2	☐3	☐4	☐5
20. I feel a sense of control over my condition	☐1	☐2	☐3	☐4	☐5
21. I feel I have a very good life despite my health problems	☐1	☐2	☐3	☐4	☐5
22. I have information to handle difficulties related to my condition	☐1	☐2	☐3	☐4	☐5
23. I have shared with others how I keep myself well	☐1	☐2	☐3	☐4	☐5
24. I have the skills that help me feel in control of my condition	☐1	☐2	☐3	☐4	☐5

	Strongly Disagree	Disagree	Neither	Agree	Strongly Agree
25. I would refuse a treatment if I thought it was not the best thing for me	☐1	☐2	☐3	☐4	☐5
26. Knowing more about my condition helps me to manage it	☐1	☐2	☐3	☐4	☐5
27. I feel useful in my daily life despite my condition	☐1	☐2	☐3	☐4	☐5
28. My own experience has increased my understanding of what it is like for other people to have this condition	☐1	☐2	☐3	☐4	☐5
29. I can talk to my doctor if I change my mind concerning my treatment	☐1	☐2	☐3	☐4	☐5
30. I know what my test results mean	☐1	☐2	☐3	☐4	☐5
31. I can live a normal life despite my condition	☐1	☐2	☐3	☐4	☐5
32. I am confident choosing among different options related to my condition with my doctor	☐1	☐2	☐3	☐4	☐5
33. I accept that I have to live with my condition	☐1	☐2	☐3	☐4	☐5
34. I find my health problems take over my life	☐1	☐2	☐3	☐4	☐5
35. I live my life one day at a time because of my condition	☐1	☐2	☐3	☐4	☐5
36. I need to know what is happening to me and why	☐1	☐2	☐3	☐4	☐5
37. I feel like I am actively involved in life despite my health problems	☐1	☐2	☐3	☐4	☐5

	Strongly Disagree	Disagree	Neither	Agree	Strongly Agree
38. I have shared my understanding of my condition with people who have similar conditions	☐1	☐2	☐3	☐4	☐5
39. I participate in decisions concerning my health care	☐1	☐2	☐3	☐4	☐5
40. I sometimes take health information to my doctor	☐1	☐2	☐3	☐4	☐5
41. I know how to handle difficulties related to my condition	☐1	☐2	☐3	☐4	☐5
42. I try to make the most of my life despite my condition	☐1	☐2	☐3	☐4	☐5
43. I understand my condition	☐1	☐2	☐3	☐4	☐5
44. I would feel able to refuse a decision made by my doctor concerning my treatment	☐1	☐2	☐3	☐4	☐5
45. I have a positive outlook towards my condition	☐1	☐2	☐3	☐4	☐5
46. People who are struggling with similar health conditions ask me for advice	☐1	☐2	☐3	☐4	☐5
47. I have all the knowledge I need to manage my condition	☐1	☐2	☐3	☐4	☐5
48. Without my health problems I could achieve more	☐1	☐2	☐3	☐4	☐5
49. I know how to control my health problems	☐1	☐2	☐3	☐4	☐5
50. I know enough about my condition	☐1	☐2	☐3	☐4	☐5
51. I feel there is purpose and meaning in my life despite my health problems	☐1	☐2	☐3	☐4	☐5

C. You and your condition(s)…

The questions below ask about how you have been feeling about your condition(s) during the **past two weeks.** Please read each statement carefully and then indicate the response that best describes the extent to which you agree or disagree

	Strongly Disagree	Disagree	Agree	Strongly Agree
1. I feel my condition is something I will never recover from	☐1	☐2	☐4	☐5
2. I feel my condition is serious, but I will be able to return to life as it was before my illness	☐1	☐2	☐4	☐5
3. I feel my condition has changed my life permanently so it will never be as good again	☐1	☐2	☐4	☐5
4. I feel that I am the same person that I was before my illness	☐1	☐2	☐4	☐5
5. I feel that my relationships with other people have not been negatively affected by my illness	☐1	☐2	☐4	☐5
6. I feel that my experience with my condition has made me a better person	☐1	☐2	☐4	☐5
7. I feel that having my condition has interfered with my achievement of the most important goals I have set for myself	☐1	☐2	☐4	☐5

D. Managing your condition(s)…

We would like to know **how confident** you feel about doing certain activities relating to managing your condition(s). For each of the following questions, please **circle** the number that corresponds to how confident you feel at the present time.

How confident are you that you can…..

1. … do all the things necessary to manage your condition on a regular basis? (Please circle **one** number only)

1	2	3	4	5	6	7	8	9	10

Not at all confident … Totally confident

2. … judge when the changes in your illness mean you should visit a doctor? (Please circle **one** number only)

1	2	3	4	5	6	7	8	9	10

Not at all confident … Totally confident

3. … do the different tasks and activities needed to manage your health condition so as to reduce your need to see a doctor? (Please circle **one** number only)

1	2	3	4	5	6	7	8	9	10

Not at all confident … Totally confident

4. … reduce the emotional distress caused by your health condition so that it does not affect your everyday life? (Please circle **one** number only)

1	2	3	4	5	6	7	8	9	10

Not at all confident … Totally confident

5. … do things other than just taking medication to reduce how much your illness affects your everyday life? (Please circle **one** number only)

1	2	3	4	5	6	7	8	9	10

Not at all confident … Totally confident

E. Your visits to your GP surgery...

Staying healthy can be difficult when you have a chronic condition. We would like to learn about the help you get in relation to your condition(s) from your health care team. This might include your regular doctor, his or her nurse, or health care assistant who treats your condition.

When I received care for my chronic condition over the past six months, I was...

	Almost never	Generally not	Sometimes	Most of the time	Almost always
1. ... asked for my ideas when we made a treatment plan.	☐1	☐2	☐3	☐4	☐5
2. ... given choices about treatment to think about.	☐1	☐2	☐3	☐4	☐5
3. ... asked to talk about any problems with my medicines or their effects.	☐1	☐2	☐3	☐4	☐5
4. ... given a written list of things I should do to improve my health.	☐1	☐2	☐3	☐4	☐5
5. ... satisfied that my care was well organised.	☐1	☐2	☐3	☐4	☐5
6. ... shown how what I did to take care of myself influenced my condition.	☐1	☐2	☐3	☐4	☐5
7. ... asked to talk about my goals in caring for my condition.	☐1	☐2	☐3	☐4	☐5
8. ... helped to set specific goals to improve my eating or exercise.	☐1	☐2	☐3	☐4	☐5
9. ... given a copy of my treatment plan.	☐1	☐2	☐3	☐4	☐5

When I received care for my chronic condition over the past six months, I was…

	Almost never	Generally not	Sometimes	Most of the time	Almost always
10. … encouraged to go to a specific group or class to help me cope with my chronic condition.	☐1	☐2	☐3	☐4	☐5
11. … asked questions, either directly or on a survey, about my health habits.	☐1	☐2	☐3	☐4	☐5
12. … sure that my doctor or nurse thought about my values, beliefs, and traditions when they recommended treatments to me.	☐1	☐2	☐3	☐4	☐5
13. … helped to make a treatment plan that I could carry out in my daily life.	☐1	☐2	☐3	☐4	☐5
14. … helped to plan ahead so I could take care of my condition even in hard times	☐1	☐2	☐3	☐4	☐5
15. … asked how my chronic condition affects my life.	☐1	☐2	☐3	☐4	☐5
16. … contacted after a visit to see how things were going.	☐1	☐2	☐3	☐4	☐5
17. … encouraged to attend programs in the community that could help me.	☐1	☐2	☐3	☐4	☐5

Over the past six months, when I received care for my chronic condition, I was…

	Almost never	Generally not	Sometimes	Most of the time	Almost always
18. … referred to a dietitian, health educator, or counselor.	☐1	☐2	☐3	☐4	☐5
19. … told how my visits with other types of doctors, like the eye doctor or surgeon, helped my treatment.	☐1	☐2	☐3	☐4	☐5
20. … asked how my visits with other doctors were going.	☐1	☐2	☐3	☐4	☐5

F. Seeing the doctor you prefer…

1. Is there a particular doctor you prefer to see at your GP surgery or health centre?

Yes ☐1

No ☐2

There is usually only one doctor in my GP surgery or health centre ☐3

2. How often do you see the doctor you prefer to see?

Always or almost always ☐1

A lot of the time ☐2

Some of the time ☐3

Never or almost never ☐4

Not tried at this GP surgery or health centre ☐5

G. Seeing a doctor in the GP surgery or health centre…

Please answer these next questions about the **last time** you saw a doctor at your GP surgery about your long-term condition(s)

1. The **last time** you saw a doctor at your GP surgery or health centre, how good was the doctor at each of the following?

	Very Good	Good	Neither	Poor	Very poor	Does not apply
a. Giving you enough time	☐1	☐2	☐3	☐4	☐5	☐6
b. Asking about your symptoms	☐1	☐2	☐3	☐4	☐5	☐6
c. Listening to you	☐1	☐2	☐3	☐4	☐5	☐6
d. Explaining tests and treatments	☐1	☐2	☐3	☐4	☐5	☐6
e. Involving you in decisions about your care	☐1	☐2	☐3	☐4	☐5	☐6
f. Treating you with care and concern	☐1	☐2	☐3	☐4	☐5	☐6
g. Taking your problems seriously	☐1	☐2	☐3	☐4	☐5	☐6

Please answer these next questions about the **last time** you saw a doctor at your GP surgery about your long-term condition(s)…

2. When you **last** saw a doctor at your GP surgery about your long-term condition, did you have confidence and trust in that doctor?

- Yes, definitely ☐1
- Yes, to some extent ☐2
- No, not at all ☐3
- Don't know/ can't say ☐4

3. When was the <u>last time</u> you visited your GP surgery to see your GP or Practice nurse?

☐☐ Month ☐☐ Year

4. Thinking about the <u>last visit</u> you made to your GP surgery, was this to see your…

GP	☐ 1
Nurse	☐ 2

Please answer these next questions about the **<u>last time</u>** you saw a doctor at your GP surgery about your long-term condition…

5. For each of the below, please tick the box that best describes how you felt <u>immediately after</u> the visit:

	Much better	Better	Same or less	Not applicable
a) Able to cope with life	☐ 1	☐ 2	☐ 3	☐ 4
b) Able to understand your illness	☐ 1	☐ 2	☐ 3	☐ 4
c) Able to cope with your illness	☐ 1	☐ 2	☐ 3	☐ 4
d) Able to keep yourself healthy	☐ 1	☐ 2	☐ 3	☐ 4

	Much more	More	Same or less	Not applicable
e) Confident about your health	☐ 1	☐ 2	☐ 3	☐ 4
f) Able to help yourself	☐ 1	☐ 2	☐ 3	☐ 4

H. About you...

Please answer the following questions. Either tick one box or write your answer in the space provided.

Please remember that all the information given will be **confidential.**

1. Are you:

Male	☐	1
Female	☐	2

2. How old are you now? ____________ years

3. Which ethnic group do you belong to? *(Please tick only one box)*

White	☐ 1		Indian	☐ 5	
Black – Caribbean	☐ 2		Pakistani	☐ 6	
Black – African	☐ 3		Bangladeshi	☐ 7	
Black – other	☐ 4		Chinese	☐ 8	
Other	☐ 9	Please specify ____________			

4. Which of these qualifications do you have? (Please tick all that apply)

1 or more O levels/CSE/GCSEs (any grade)	☐	1
1 or more A levels or AS levels	☐	2
Degree	☐	3
NVQ	☐	4
Other trade qualification	☐	5
A professional qualification	☐	6
No qualifications	☐	7

5. Which of these best describes your current work situation?
(Please tick all that apply)

In paid work (full or part-time including self-employed) ☐ 1
Unemployed ☐ 2
Retired from paid work ☐ 3
Unable to work because of long-term disability or ill health ☐ 4
Looking after the family or home ☐ 5
In full-time education or training ☐ 6
Voluntary work ☐ 7
Other ☐ 8

If other please describe:

6. Is your accommodation: (Please tick one box only)

Owner-occupied/mortgaged ☐ 1
Rented from local authority/housing association ☐ 2
Rented from a private landlord ☐ 3
Other arrangements ☐ 4

If other please describe:

About you...

7. How long have you been seeing the doctor/nurse with this condition?
   (If you are unsure, please estimate in the box below)

☐☐ Years

8. In general, how would you rate your health?

Excellent	☐ 1
Very good	☐ 2
Good	☐ 3
Fair	☐ 4
Poor	☐ 5

Any Comments?

The space below is for any comments you may have regarding the pilot survey you have just completed

Please return the questionnaire in the enclosed prepaid envelope provided (no stamp is required).

Thank you very much for taking part.
